# Textbook Outcome of Laparoscopic Microwave Ablation for Hepatocellular Carcinoma

**DOI:** 10.3390/cancers15020436

**Published:** 2023-01-10

**Authors:** Jacopo Lanari, Silvia Caregari, Ilaria Billato, Enrico Gringeri, Francesco D’Amico, Giancarlo Gemo, Domenico Bassi, Francesco Enrico D’Amico, Riccardo Boetto, Alessandra Bertacco, Andrea Marchini, Sara Lazzari, Marco Brolese, Mattia Ballo, Alessandro Vitale, Umberto Cillo

**Affiliations:** 1 Department of Surgical, Oncological and Gastroenterological Sciences (DiSCOG), University of Padua, 35128 Padua, Italy; 2 General Surgery 2-Hepato-Pancreato-Biliary Surgery and Liver Transplantation Unit, Padua University Hospital, 35128 Padua, Italy; 3 Department of Biology (DiBio), University of Padua, 35128 Padua, Italy

**Keywords:** hepatocellular carcinoma, laparoscopic, microwave ablation, textbook outcome

## Abstract

**Simple Summary:**

Textbook outcome (TO) is a novel composite measure that provides a comprehensive evaluation of a specific treatment which can be useful for procedures’ standardization and reliable comparisons between different centers. This tool is gaining growing interest and widespread importance in many different fields. Considering liver surgery, however, an agreement was reached, and TO assessment was evaluated on a large scale only concerning liver resection. This study aimed to investigate the first TO for laparoscopic microwave ablation for hepatocellular carcinoma. Furthermore, the current study investigated the expendability of this tool for prognostic purposes.

**Abstract:**

In the context of spreading interest in textbook outcome (TO) evaluation in different fields, we aimed to investigate an uncharted procedure, that is, laparoscopic microwave ablation (MWA) for hepatocellular carcinoma (HCC). Absence of post-MWA complications, a hospital stay of three days, no mortality nor readmission within 30 days, and complete response of the target lesion at post-MWA CT scan defined TO achievement. Patients treated between January 2014 and March 2021 were retrospectively reviewed, and of the 521 patients eligible for the study, 337 (64.7%) fulfilled all the quality indicators to achieve the TO. The absence of complications was the main limiting factor for accomplishing TO. At multivariable analysis, Child–Pugh B cirrhosis, age of more than 70 years old, three nodules, and MELD score ≥ 15 were associated with decreased probabilities of TO achievement. A score based on these factors was derived from multivariable analysis, and patients were divided into three risk groups for TO achievement. At survival analysis, overall survival (OS) was significantly (*p* = 0.001) higher in patients who achieved TO than those who did not. Moreover, OS evaluation in the three risk groups showed a trend coherent with TO achievement probability. The present study, having assessed the first TO for laparoscopic MWA for HCC, encourages further broader consensus on its definition and, on its basis, on the development of clinically relevant tools for managing treatment allocation.

## 1. Introduction

Textbook outcome (TO) is a composite measure that captures the most desirable outcomes of a specific treatment as a single indicator. This tool is fundamental in the surgical field because treatment outcome is a complex synthesis of multiple variables such as peri-operative factors, postoperative morbidity, and oncological radicality (R0). In the current era, TO is becoming a spreading tool to measure the quality of surgical procedures in multiple specialties such as hepatic, pancreatic, head and neck, lung [1,2,3,4,5], and even transplantation [6,7,8,9] surgery. TO is functional for procedures’ standardization and to weigh their cost-effectiveness considering tailored medicine. Moreover, it allows for comparing different centers’ activities in a more reliable and standardized fashion.

Concerning liver surgery, an international agreement about the definition of TO for resection, both in a laparoscopic and open setting, was recently investigated and reached [10]. Furthermore, its incidence in an extensive international multicenter database was assessed [11].

Serra et al. [12] published the first TO for percutaneous radiofrequency ablation (RFA) for hepatocellular carcinoma (HCC), showing that moderate comorbidities, Eastern Cooperative Oncology Group (ECOG) Performance Status (PS), postoperative complications, number and diameter of nodules were the variables critical for TO achievement.

Given our remarkable experience with laparoscopic microwave ablation (MWA) for HCC [13], with the present study, we aim to investigate the first TO for this minimally invasive surgical procedure.

## 2. Materials and Methods

This is a retrospective analysis from a prospectively collected database of HCC patients treated with laparoscopic MWA between January 2014 and March 2021 at General Surgery 2-Hepato-pancreato-biliary Surgery and Liver transplantation Unit, Padua University Hospital, Padua, Italy.

Patients included in the study were those undergoing laparoscopic MWA for HCC, either for de novo, treatment-naïve HCC, or for recurrent cases after liver resection (LR) or other treatments. For this study, the exclusion criteria were HCC outside Milan criteria and patients with ECOG PS > 1.

**Figure 1:**
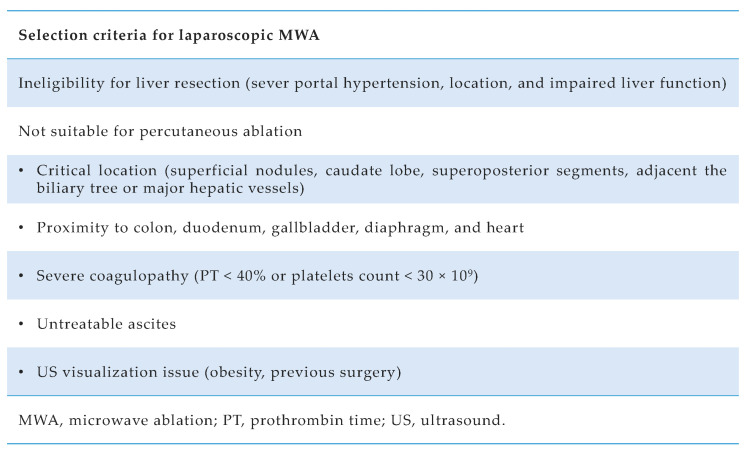
Selection criteria for laparoscopic microwave ablation in hepatocellular carcinoma (HCC) patients at Padua University Hospital.

Our center’s selection criteria for laparoscopic MWA were previously described [13], in addition to the surgical procedure [14]. In particular, the laparoscopic approach is indicated when percutaneous is not feasible (Figure 1). A 14-G water-cooled coaxial antenna was inserted into the tumor under US guidance. All MWA utilized a 2.45 MHz microwave generator (AMICA GEN; HS Hospital Service SpA, Aprilia, Italy) with power settings at a median of 40 W (interquartile range (IQR), 30–60 W). 

Tumor burden (number and dimension of HCC nodules) was measured at the last CT scan or MRI before ablation. In addition, the Adult Comorbidity Evaluation-27 (ACE-27) [15] was used to classify patients’ comorbidities other than cirrhosis.

Laparoscopic MWA complications were censored within 30 postoperative days; complications were recorded as follows: fever (requiring prolongation/change in antimicrobial therapy), nausea and vomiting, pleural effusion (if treated with albumin infusion and diuretic therapy or thoracentesis), pneumothorax (if pleural drainage was needed), ascites (requiring albumin supplementation or diuretics), hemoperitoneum and liver function impairment according to the 50-50 criteria [16]. In addition, the Clavien–Dindo classification [17] was used to grade post-operative complications. 

Post-MWA mortality was censored in case of mortality during the hospitalization or within 30 days after the procedure. Readmission due to treatment-related complications of any type was edited within 30 days after the MWA. Prolonged length of stay (LOS) was censored if discharge occurred after the cohort’s 75th percentile of the LOS.

Contrast-enhanced CT or MRI was repeated one month after MWA to assess the efficacy of ablation: the disappearance of any intra-tumoral arterial enhancement in the target lesion/s defined complete response (CR) [18,19].

The analysis was designed per patient rather than per procedure. For each patient, only the first MWA procedure was considered, excluding re-treatments on previous MWA, due to incomplete ablation diagnosed at one month by contrast-enhanced CT scan.

As recently proposed for percutaneous procedures [12], the TO was defined as follows: absence of post-MWA complications, a hospital stay of three days, no mortality nor readmission within 30 days, and complete response of the target lesion at 1-month post-MWA CT scan.

The present study was conducted in compliance with regional ethics committees and national laws of the participating institution: no patient approval was needed due to the study’s retrospective design. Patients gave written consent for every procedure performed in the hospital, including data for medical purposes, which were obtained consistent with the Declaration of Helsinki. All procedures were performed in accordance with the Declaration of Istanbul. No one received compensation or was offered any incentive for participating in this study. 

### Statistical Analysis

Values for categorical variables were expressed as totals and percentages, whereas values for continuous variables were expressed as medians and interquartile ranges (IQR). Statistical analyses were performed using Pearson’s chi-squared test or Fisher’s test for categorical variables and the Kruskal–Wallis rank sum test for continuous variables.

The length of follow-up was calculated from the surgery date to the patient death (overall survival—OS) or the latest follow-up. The duration of follow-up and survival was expressed as median (interquartile ranges). Survival curves were calculated using the Kaplan–Meier technique and compared with the log-rank test.

Simple and multivariable logistic regression was used to investigate the potential predictors of TO achievement. Stepwise selection (backward elimination) was used to compute multivariable regression analyses. A *p*-value < 0.05 indicated statistical significance; variables with a *p*-value < 0.1 were considered marginal statistical significance. Statistical analyses were performed using R, RStudio 4.2.1 (2022).

## 3. Results

From 1 January 2014, to 31 March 2021, 826 patients underwent laparoscopic MWA for HCC at our center. Two hundred and sixty-seven patients had HCC outside Milan criteria, and thirty-eight scored ECOG PS > 1. Once exclusion criteria were applied, 521 patients were eligible for the study. Four hundred and thirty-nine (84%) patients were male, and the median age was 63.3 years old (IQR 56.9–68.1). Mild comorbidities were present in 53% (*n* = 274) of patients and moderate in 5.4% (*n* = 28). Comorbidities other than chronic hepatitis/cirrhosis were absent in 42% (*n* = 216) of the whole cohort, whereas severe comorbidities were absent. Patients graded as ECOG PS 1 were 60 (12%). Hepatitis C virus (HCV) post-necrotic cirrhosis (PNC) was the primary underlying etiology, and it was present in 246 (47%) patients. One hundred and twelve patients (26%) were classified as Child–Pugh B, and 31 patients (6.2%) had a MELD score ≥15; portal hypertension was present in 55% of the whole cohort. Two hundred and sixty-four (51%) patients had de novo, treatment-naïve HCC/s. A single tumor was treated in 265 (51%) patients, and the maximum diameter of the target lesion was <2 cm in 260 (50%) patients. Clinical and perioperative characteristics are listed in Table 1.

Two patients (0.4%) died within 30 days after the procedure. Five (1%) patients developed a severe complication (Clavien–Dindo ≥ 3b), whereas the majority had no complications (*n* = 398; 76.4%). The most common mild complication observed was post-operative ascites (*n* = 61; 11.7%). The median LOS was two days (IQR: 1–3); consequently, discharge after three days was considered a prolonged hospital stay. The readmission rate was 2.30% (*n* = 12). Finally, CT-scan at one month showed CR of the target lesion/s in 491 (94.2%) patients; only 30 (5.8%) failed to achieve CR at one month CT-scan after MWA. Finally, 162 (31.1%) patients were disease-free at the end of the follow-up (Table 2).

### 3.1. TO Achievement, Determinants, and Risk Groups

**Figure 2:**
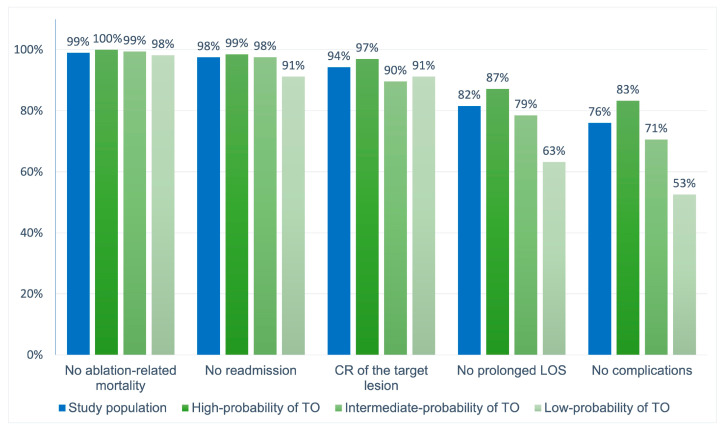
The proportion of patients who achieved each desired health outcome forming the textbook outcome (TO) in the whole population, and in the three risk groups individually.

A total of 337 (64.7%) patients fulfilled all the parameters to achieve the TO. Achievement of each TO item was calculated separately with cumulative percentages to identify which indicator was the main limiting factor for accomplishing TO. The absence of complications is the item found to have the lowest achievement incidence compared to the others (76%) (Figure 2).

Moreover, simple and multivariable logistic regression for determinants of TO achievement was performed, and the results are listed in Table 3. 

As a result of the multivariable analysis, probabilities of TO achievement decreased in patients with Child–Pugh B cirrhosis (comparator: Child–Pugh A; OR: 0.28; 95% CI: 0.17–0.46; *p* < 0.001), patients more than 70 years old (comparator: age < 70; OR: 0.49; 95% CI: 0.27–0.88; *p* = 0.017), patients with three nodules (comparator: 1 nodule; OR: 0.54; 95% CI: 0.32–0.93; *p* = 0.025) and MELD score ≥ 15 (comparator: MELD < 15; OR: 0.44; 95% CI: 0.17–1.09; *p* = 0.086). The presence of mild or moderate comorbidities other than chronic hepatitis/cirrhosis showed no influence on TO achievement.

**Figure 3:**
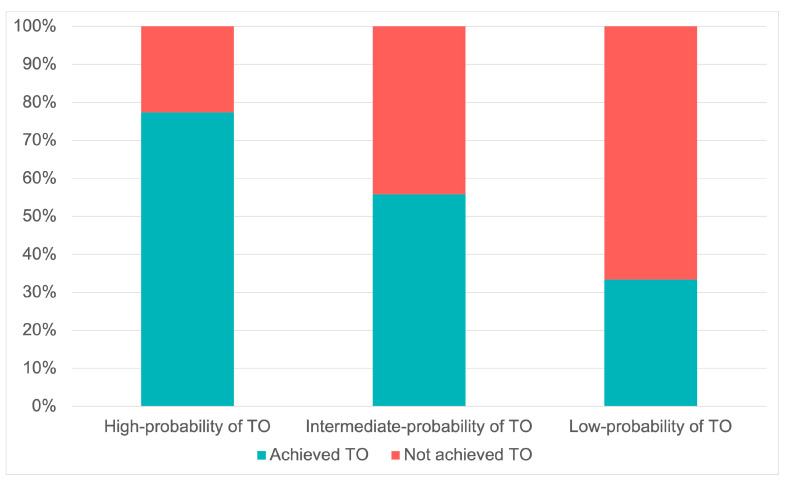
The proportion of textbook outcome (TO) achievement related to the three risk groups identified.

At a further analysis, the highest OR value from the multivariable model was used as the lowest common denominator to derive risk points for TO determinants, which were consequently assigned as follows: age ≥ 70, 2 points; MELD ≥ 15, 2 points; 3 nodules, 2 points; Child–Pugh B, 1 point. Three risk groups were then identified based on the cumulative score reached by each patient: patients who scored 0 had a high probability of TO achievement (77.3% TO achievement), those who scored 1–2 had an intermediate-probability of TO achievement (55.8% TO achievement), and those who scored ≥ 3 had low-probability of TO attainment (33.3% TO achievement) (Figure 3). Moreover, Figure 1 shows the accomplishment of every quality indicator forming the TO considered individually in each risk group.

### 3.2. Survival Analysis

**Figure 4:**
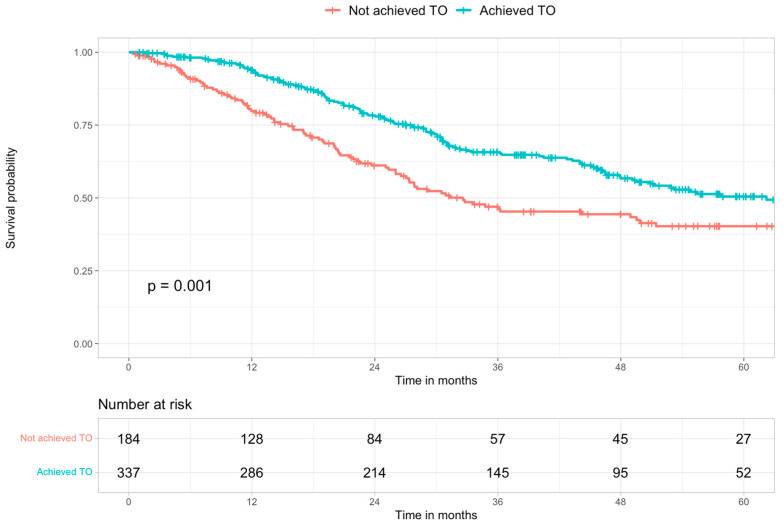
Kaplan–Meier survival curves of the study population stratified according to textbook outcome (TO) achievement.

The median follow-up was 28.7 months (IQR: 14.2–49.9), and the median survival was 51.3 months (95% CI: 45.8–67.1) in the study population. The median survival in the cohort of patients who achieved TO was 62.2 months (95% CI: 49.5–not reached) compared to 32.6 months (95% CI: 27.1–51.4) in the cohort of patients who did not. The OS of patients who achieved TO was 93.9%, 65.7%, and 50.5% at 1, 3, and 5 years, respectively, compared to 1-, 3- and 5-year OS of patients who did not achieve TO that was 79.8%, 47%, and 40.3%, respectively (*p* = 0.001) (Figure 4).

**Figure 5:**
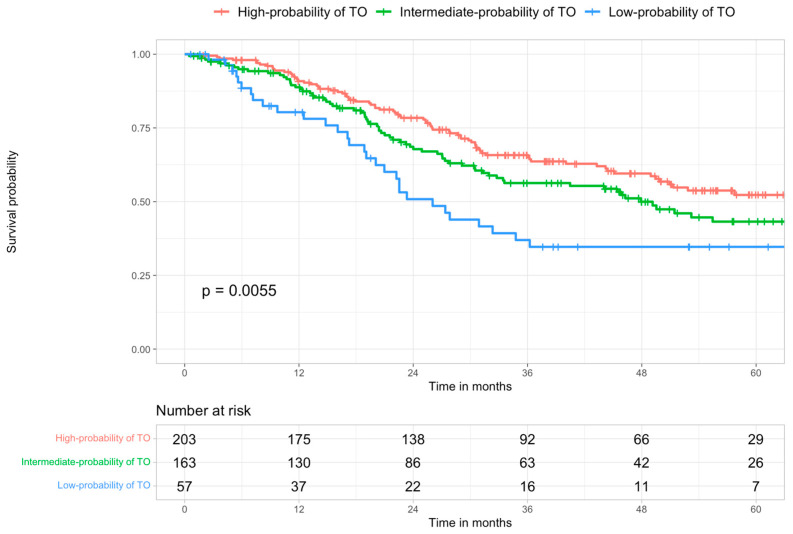
Kaplan–Meier survival curves of the study population stratified according to the three risk groups identified. High vs. intermediate probability *p* = 0.0827; high vs. low probability *p* = 0.0039; intermediate vs. low probability *p* = 0.1170.

When survival analysis was performed, taking into account the risk groups stratification, the highest OS (90.9%, 65.8%, and 52.3% at 1-, 3- and five years, respectively), was observed in the group with the highest probability of TO achievement, with a progressive and significant (*p* = 0.0055) OS reduction, consensual to the decreasing of TO achievement probability (80.3%, 36.9% and 34.7% at 1-, 3- and 5-years, respectively, in the low-probability group) (Figure 5).

## 4. Discussion

Individual outcome variables represent the conventional tools for evaluating healthcare quality. However, TO was recently introduced as a composite measure to capture the multidimensional aspect of the care pathway and is progressively spreading and gaining importance in various surgical fields. For example, in hepato-pancreato-biliary (HPB) surgery, given the complex procedures and patients involved, TO development is considered of primary importance, as recently demonstrated by Pretzsch et al., who systematically reviewed 30 papers on this topic [20].

One of the main issues with the TO definition is the item selection. The ones we used in our study are based on the choice made by Serra et al. [12] in their similar analysis of percutaneous RFA and recall the ones formerly suggested by Merath et al. [21]. The limit of this choice is the need for a broad consensus for the definition of these criteria, which, based on the opinion of a small group of experts, are at increased risk of individual bias. Nevertheless, items were chosen based on what is commonly considered a surrogate of safety (i.e., postoperative complications, LOS, and the need for readmission) and efficacy (i.e., response to treatment) so that a consensus could be expected even in the absence of a rigorous methodology. 

While for ablation therapy, an international agreement about TO definition criteria needs to be improved, advances have been recently made in the field of LR. Görcec et al. [10,11] made a cohesive effort to provide an international expert consensus-based definition of TO in liver surgery (TOLS). A panel of 44 expert surgeons, through a modified 4-round Delphi process, defined TOLS as the absence of intraoperative grade ≥ 2 incidents (described according to the Oslo classification [22]), postoperative bile leak of grade B or C (according to the severity grading of the International Study Group of Liver Surgery [23]), postoperative liver failure grade B or C (according to the severity grading of the International Study Group of Liver Surgery [24]), major postoperative complications within 90 days (Clavien–Dindo grade III or higher [25]), readmission within 90 days after discharge due to surgery related major complications (Clavien–Dindo Grade III or higher), in-hospital or 90-day mortality and the presence of R0 resection margin (i.e., 1 mm or more tumor-free margin). 

One of the main differences between the TO items used in the current study and the ones established by Görcec et al. is the length of hospital stay as a parameter of evaluation. Although a consensus about LOS duration could not be reached in an international setting, the assessment of this aspect was considered significant by Görcec et al., so they suggested the development of an extended definition of TOLS including LOS (TOLS+) to be used on a national level. Another difference between the current study’s TO items and internationally established ones is how complications, readmissions, and mortality were evaluated at 30 days and 90 days, respectively. A more extended observation period was chosen in TOLS based on studies proving that shorter periods were inadequate for evaluation due to their incomplete representativeness [26,27]. However, the magnitude of MWA is undoubtedly minor compared to LR and, consequently, its short-term effect on patient morbidity; hence, a shorter observation period seems justifiable. 

In addition to the high attention reserved for TO in HPB surgery, it was still missing in this particular field of liver surgery. To our knowledge, this is the first study on the TO of laparoscopic MWA for HCC. Our choice to base the current analysis on a comparison with the one published by Serra et al. [12] was due to the treatment technique considered. Since that was the first and only study developing a TO in the field of ablative therapy for HCC, we decided to use the same TO items, thus obtaining comparable results. 

TO achievement rate was as good as 64.7%. Indeed, the TO achievement rate in general HPB surgery ranged between 15.8% and 69.1% [20]. On the other hand, Serra et al. reported 50.3% TO achievement, with CR of the target lesion being the limiting factor. Concerning the laparoscopic procedure, quite the opposite, CR was the most frequently achieved item while TO achievement was limited by the onset of complications, probably reflecting a worse hepatic function in this population (26% Child B patients in the current study vs. 16.2% in Serra et al.). These results reinforce our previous findings showing the superiority of laparoscopic MWA over laparoscopic RFA [14] in terms of recurrence profile, most likely due to the already demonstrated technical advantages of MWA. In particular, microwave induces tumor necrosis within a shorter period of ablation, does not result in tissue desiccation or charring, achieves a more significant zone of intra-tumoral thermal injury, and has an attenuated heat sink effect [28,29]. Those factors contributed to the current popularity of percutaneous MWA. Notably, even if a slight difference in median overall population survival was observed (i.e., 60.6 months in the paper by Serra et al. and 51.3 months in the current study), when considering only the TO achieving population, median survivals were comparable, respectively, 63.5 months for percutaneous RFA and 62.2 months for laparoscopic MWA.

These findings prompt the development of further studies that could demonstrate the non-inferiority of laparoscopic procedure, especially in the current era of therapeutic hierarchy for HCC treatment allocation [30,31], since it has already been shown that laparoscopic technique can overcome many limitations of percutaneous one (i.e., critical locations, proximity to other organs, severe coagulopathy, ascites, US visualization issues) [13,32,33,34]. As previously observed, indeed, the use of this surgical approach, also in comparison with other techniques such as transcatheter arterial chemoembolization (TACE) and liver transplantation, even if not strictly established and recommended by guidelines, could be justifiable in terms of results and survival, widening the audience of patients who can undergo potentially curative treatment [35,36]. 

Looking at risk stratification based on the parameters that impact the probability of TO achievement, our study showed concordance between a higher chance of TO achievement and better long-term results in terms of OS.

Serra et al. [12] found that moderate comorbidities, ECOG PS 1, and tumor burden (>2 nodules and nodules > 3 cm) had a negative impact on TO. We partially confirmed the previous findings: our study showed that age ≥ 70 years, MELD ≥ 15, Child B, and three nodules were associated with decreased probability of TO achievement.

Remarkably, all the characteristics proposed so far are evaluable in pre-operative settings, suggesting the development of tools for the pre-operative evaluation of the best treatment allocation for the patients based on their characteristics. Considering the previously described results, we can see the ideal candidate for MWA: relatively young, with preserved liver function and few HCC nodules. Furthermore, those patients have a higher probability of TO achievement and long-term survival. Notably, five years OS in our cohort of patients who did not achieve TO and who had a low likelihood of TO achievement was 40.3% and 34.7%, respectively, which are not poor results considering the characteristics of those patients (i.e., more frequently old, high MELD, Child B, multinodular). Indeed, TO is not a parameter to exclude patients from treatment when only a few therapeutic alternatives are available with poor outcomes (most likely, TACE). Consequently, tools derived from the TO analysis can help clinicians to allocate each patient to the best treatment based on the expected outcome, as dictated by therapeutic hierarchy, in the context of a multiparametric and multidisciplinary approach. 

The present study has several limitations. First, it includes data from a single center activity; additionally, although prospectively collected, these data were retrospectively analyzed. Nonetheless, the definition of TO items for liver ablation procedure cannot be set only by these sparse experiences, and a comprehensive agreement is desirable. Hence, we advocate for further consensus by experts in the field and more robust evidence from multicentric studies. Once a widely accepted instrument is obtained, it will be helpful and informative for comparing the results obtained by different centers.

## 5. Conclusions

In conclusion, with the present study, we investigate the first TO for laparoscopic MWA for HCC based on a single institution experience, encouraging further broader consensus on its definition and, on its basis, on the development of clinically relevant tools for the management of treatment allocation.

## Figures and Tables

**Table 1:** Clinical and perioperative characteristics.

Characteristic	Not Achieved TO ^1^ *n* = 184	Achieved TO ^1^ *n* = 337	*p* ^2^
**Sex (male)**	149/184 (81%)	290/337 (86%)	0.13
**Age** (y)	64.5 (57.9–69.15)	62.9 (56.6–67.8)	**0.092**
**Comorbidities**			
- None	72/183 (39%)	144/335 (43%)	**0.038**
- Mild	98/183 (54%)	176/335 (53%)	
- Moderate	13/183 (7.1%)	15/335 (4.5%)	
**ECOG PS = 1**	24/184 (13%)	36/337 (11%)	0.42
**Liver disease etiology**			
- HCV	93/184 (51%)	153/337 (45%)	0.26
- HBV	36/184 (20%)	74/337 (22%)	0.52
- Alcohol	56/184 (30%)	104/337 (31%)	0.92
**Platelet count** (×10^3^/mL)	85 (64.5–130)	95 (67.8–141.5)	**0.031**
**Portal hypertension**	113/184 (61%)	171/337 (51%)	**0.019**
**MELD ≥ 15**	21/180 (12%)	10/318 (3.1%)	**<0.001**
**Child–Pugh Classes**			
- Child A	92/159 (58%)	229/274 (84%)	**<0.001**
- Child B	67/159 (42%)	45/274 (16%)	
**Previous HCC treatments**	91/184 (49%)	166/337 (49%)	0.97
**Number of nodules**			
- 1	93/184 (51%)	172/337 (51%)	**0.015**
- 2	42/184 (23%)	107/337 (32%)	
- 3	49/184 (27%)	58/337 (17%)	
**Diameter of the largest nodule** (cm)			
- <2	75/184 (41%)	185/337 (55%)	**0.003**
- 2–3	54/184 (29%)	90/337 (27%)	
- 3–5	55/184 (30%)	62/337 (18%)	
**pRBC**	10/183 (5.5%)	1/331 (0.3%)	**<0.001**
**pFFP**	24/183 (13%)	5/331 (1.5%)	**<0.001**

^1^ Median (IQR); *n*/N (%); ^2^ Wilcoxon rank sum test; Pearson’s chi-squared test; Fisher’s exact test ECOG PS, Eastern Cooperative Oncology Group Performance Status; HCV, hepatitis C virus; HBV, hepatitis B virus; MELD, model for end-stage liver disease; pRBC, patients transfused with packed red blood cell; pFFP, patients transfused with fresh frozen plasma.

**Table 2:** Postoperative outcomes.

Characteristic	N (%)
Fever	30 (5.8%)
Nausea and vomiting	1 (0.2%)
Pleural effusion	4 (0.8%)
Pneumothorax	3 (0.6%)
Ascites	61 (11.7%)
Hemoperitoneum	1 (0.2%)
Liver disfunction (50-50 criteria)	31 (6.0%)
30 days mortality	2 (0.4%)
Readmission	12 (2.3%)
LOS > 3 days	96 (18.4%)
No CR	30 (5.8%)

LOS, length of hospital stay; CR, complete response.

**Table 3:** Simple and multivariable logistic regression for determinants of TO achievement.

	Univariable Analysis			Multivariable Analysis		
Variables	OR ^1^	95% CI ^2^	*p*	OR^1^	95% CI ^2^	*p*
**Sex (male)**	1.45	0.89, 2.34	0.13			
**Age ≥ 70**	0.74	0.46, 1.22	0.2	0.49	0.27, 0.88	**0.017**
**Comorbidities**						
- Mild	0.90	0.62, 1.31	0.6			
- Moderate	0.58	0.26, 1.29	0.2			
**ECOG PS = 1**	0.80	0.46, 1.40	0.4			
**HCV**	0.81	0.57, 1.17	0.3			
**HBV**	1.16	0.74, 1.82	0.5			
**Alcohol**	1.02	0.69, 1.51	>0.9			
**INR**	0.12	0.04, 0.35	**<0.001**			
**Bilirubin level** (mg/dL)	0.64	0.49, 0.80	**<0.001**			
**Portal hypertension**	0.65	0.45, 0.93	**0.020**			
**MELD ≥ 15**	0.25	0.11, 0.52	**<0.001**	0.44	0.17, 1.09	**0.086**
**Child–Pugh class B**	0.27	0.17, 0.42	**<0.001**	0.28	0.17, 0.46	**<0.001**
**Number of nodules**						
- 2	1.38	0.89, 2.15	0.2	1.41	0.85, 2.35	0.2
- 3	0.64	0.41, 1.01	**0.055**	0.54	0.32, 0.93	**0.025**
**Portal hypertension**	0.65	0.45, 0.93	**0.020**			
**Diameter largest nodule** (cm)						
- 2–3	0.66	0.43, 1.02	**0.061**			
- 3–5	0.46	0.29, 0.72	**<0.001**			
**pRBC**	0.05	0.00, 0.28	**0.005**			
**pFFP**	0.10	0.03, 0.25	**<0.001**			

^1^ OR = odds ratio (<1 = lower probability of TO achievement; ≥1 = higher probability of TO achievement); ^2^ CI = confidence interval. ECOG PS, Eastern Cooperative Oncology Group Performance Status; HCV, hepatitis C virus; HBV, hepatitis B virus; INR, international normalized ratio; MELD, model for end-stage liver disease; pRBC, patients transfused with packed red blood cell; pFFP, patients transfused with fresh frozen plasma.

## Data Availability

The data presented in this study are available on request from the corresponding author. The data are not publicly available due to privacy restrictions according to Italian law.
